# Predictive factors of in-hospital mortality in very old patients following rapid response team activation

**DOI:** 10.1186/cc14686

**Published:** 2015-09-28

**Authors:** Henrique Palomba, Andréia Pardini, Antonio Capone Neto, Felipe M de T Piza, Michele Jaures, Thiago D Corrêa

**Affiliations:** 1 Hospital Israelita Albert Einstein, Morumbi, São Paulo, SP, Brazil

## Introduction

The number of very old patients (80 years old) with chronic illness and functional impairment requiring major medical attention is increasing. Rapid Response Teams (RRT) were implemented to improve the recognition and response to deteriorating ward patients. However, the characteristics and outcomes of very old patients assisted by RRT during hospitalization are not well described.

## Objective

The primary objective was to evaluate the rate, characteristics and outcomes of very old patients (80 years old) assisted by RRT during hospitalization.

## Methods

A total of 538 very old patients assessed by RRT between January 2012 and December 2013 was included in this analysis. A multivariate logistic regression analysis was undertaken to address which predictors were associated with increased in-hospital mortality. Early RRT call was defined as RRT activation within 48 hours from hospital admission and late RRT call if it happened afterwards.

## Results

From January 2012 and December 2013, 2072 patients were assisted by the RRT. Very old patients (age ≥80 years) accounted for 26 % (538/2072) of patients. The mean (SD) age was 87 (4.8) years and 42 % (*n *= 224) were male. There were 193 (36 %) early RRT calls and 345 (64 %) late RRT calls. The main reasons for RRT activation were respiratory failure in 35 % (*n *= 186) and mental status changes in 15 % (*n *= 80). The distribution of RRT activation was uniform over the 24-hour period, with 55 % (*n *= 298) of calls during the day (7:00 a.m.-7:00 p.m.) and 45 % (*n *= 240) overnight (7:00 p.m.-7:00 a.m.). A total of 153 patients (28 %) were admitted to the ICU and the overall mortality rate was 21.5 % (110/511). Late RRT call (OR 1.73; 95 % CI 1.07-2.80; *p *= 0.025) and acute changes in peripheral oxygen saturation below 90 % (OR 1.56; 95 % CI 1.01-2.40; *p *= 0.044) were associated with increased risk of in-hospital death while admission to a step-down unit (OR 0.49; 95 % CI 0.26-0.92; *p *= 0.026) was associated with a decreased risk of in-hospital death.

## Conclusion

Multiple factors relating to the nature of RRT activation and very old patient characteristics are associated with in-hospital mortality. This information may be useful for risk stratification and determination of an appropriate treatment strategy for very old patients during hospitalization.

